# Design and implementation of a longitudinal ambulatory clerkship in the first-year curriculum at the Johns Hopkins School of Medicine

**DOI:** 10.3402/jchimp.v1i1.7033

**Published:** 2011-05-09

**Authors:** Rosalyn Stewart, Sharon Dlhosh, Christine Marino, Patricia Thomas, Maura J. McGuire

**Affiliations:** 1 Department of Medicine, Johns Hopkins University School of Medicine, Baltimore, MD, USA; 2 Johns Hopkins Community Physicians and Department of Medicine, Johns Hopkins University School of Medicine Baltimore MD, USA

**Keywords:** health professional education, education reform, primary care, faculty development

## Abstract

In response to calls for medical education reform we designed and implemented a new Longitudinal Ambulatory Clerkship (LAC) at the Johns Hopkins University School of Medicine. The LAC provides first-year medical students with their initial exposure to clinical medicine during a 12-month experience consisting of weekly clinic sessions with a practicing physician-mentor (preceptor) and longitudinal experience with a population of patients. The LAC allows students to experience firsthand many of the personal, professional, and organizational issues that impact the practice of medicine. This paper reviews the rationale, development, and challenges during implementation of this clerkship.

Medical education reform has called for early immersion of students into clinical practice to tie clinical learning to scientific learning and for integration of biomedical, scientific, and social curricula in a supportive environment that promotes professional formation (1, 2). In response to these reform concepts and as part of a decade-long internal dialoge to improve our educational processes, the Johns Hopkins University School of Medicine implemented its new ‘Genes to Society’, curriculum (GTS) in 2009 (3). The GTS included a new Longitudinal Ambulatory Clerkship (LAC) to involve first-year medical students in the practice of clinical medicine, expose them to social and behavioral aspects of health care, and ground these experiences in classroom learning.

## Background and implementation challenges

Prior to the LAC, our first-year students participated in an ‘Introduction to Medicine’ (IM) course and a clinical skills (CS) curriculum. The IM was a shadowing experience, lasted 10 weeks, and involved a heterogeneous group of faculty. The CS was taught over three 9-week quarters in small groups; the first two quarters were taught on campus by full-time faculty, while the third quarter (CSQ3) was taught by faculty in community hospitals and practices. Ambulatory care education was provided during basic pediatrics and emergency medicine clerkships and in a required Ambulatory Clerkship in Medicine (ACIM) that lasted 4 weeks, included 20 practice sessions, and targeted third- and fourth-year students. In restructuring the 4-year Hopkins curriculum, it was determined that the IM, most of CSQ3 and ACIM would be replaced by the LAC.

Developing an ambulatory clerkship experience for the first-year curriculum presented challenges in student preparation, faculty development, and curriculum integration. To address student preparation, the CS curriculum was condensed into an intensive Clinical Foundations (CF) course given in the first 4 months of medical school. Some parts of pre-ward training, like certification in cardiopulmonary life support and standard precautions, were moved to the first-year curriculum. To address professional growth of early learners we worked with a special group of advising faculty (Colleges Faculty) recently formed by our institution (4). Finally to recognize pedagogical needs of first-year clerks and the goal of close mentorship during the clerkship, we decided on a one-to-one staffing ratio of preceptors to students.

Recruiting and developing 120 qualified faculty members to staff the new clerkship presented more significant challenges. The existing ambulatory faculty, who taught third and fourth year clerkships, voiced concerns about working with inexperienced first-year students in busy office practices, as well as the yearlong teaching commitment. Globally our institution had barely enough ambulatory faculty to meet the teaching demands of the old curriculum and in the new curriculum the majority would continue previous commitments. This was particularly true during transition years, when clinical students who hadn't received LAC needed to take the ACIM as LAC was being implemented. As we looked to recruit a new faculty base for the LAC, we heard from many volunteer community hospital and faculty leaders that the economic stressors affecting primary care would make it difficult to staff a first-year clerkship unless financial support was provided. Finally, standardizing teaching, developing a diverse community faculty, and providing periodic reinforcement of our school's CS curriculum needed attention. To address these issues, we developed progressive learning goals appropriate to first-year students, worked with CF faculty to provide periodic skills reinforcement, secured a teaching stipend and CME benefits for preceptors, expanded our faculty recruiting base, and created a learning passport system to provide structured activities if regular preceptors were unavailable.

We ultimately recruited more than 160 new faculty members from community hospitals and primary care practices to staff this clerkship: 84.9% are members of our community-based (part-time) faculty and 84.9% practice primary care. Of the community faculty, 20.4% worked in practices based at affiliated hospitals and 79.6% worked in community practices. We also recruited a group of full-time faculty, primarily in surgical and medical subspecialties, to staff our passport activities. Because our faculty work in practices up to 25 miles from the main campus, we created an online faculty development program with quarterly sessions to provide training and discussion; 87.7% of our faculty have completed at least one faculty development session. The engagement of new faculty and community practices with our institution has broadened our student's collective experience of the diversity of health care delivery systems and provides a broad platform for practice-based learning and our biopsychosocial curriculum.

## Clerkship structure

### General organization

The Longitudinal Clerkship (LC) begins in the fourth month of medical school year one, lasts 12 months and enrolls all 120 medical students who work one-to-one with a practicing physician mentor (preceptor) in an ambulatory care setting. Students complete 24 half-day long ambulatory sessions with preceptors, where they see and examine patients and practice CS. As much as possible, students are expected to see patients who reflect systems and conditions being studied in scientific classroom sessions.

To consolidate and review the diverse clinic experiences of our students, they convene in small group sessions approximately every fourth week during the clerkship. Because our community preceptors may model different clinical skills than those taught in our CF curriculum, all small groups include time to review clinical skills with full-time faculty and/or with physical-exam teaching assistants (PETAs). The learning objectives for the LAC are shown in Table 1 and the general curriculum for the LAC is shown in Table 2; ambulatory topics are reviewed in 8 hours of required lecture.

**Table 1:** Learning objectives in longitudinal ambulatory clerkship

By the end of the clerkship the student will have:
1. Gained practical experience in ambulatory care by practicing with a physician mentor (preceptor) for 4 hours each week for one. academic year and by personally interviewing or examining at least two patients per session.
2. Described how important social/behavioral issues impact health care using reflection.
3. Demonstrated they can identify a chief complaint, perform a focused history and exam, and generate a SOAP note with a problem list and differential diagnosis.
4. Demonstrated proficiency in completing a standard medical case write-up.
5. Demonstrated they have followed at least one patient longitudinally during the clerkship.
6. Gained experience in self-directed learning and professionalism.

**Table 2:** Core curriculum of the longitudinal clerkship

Clinical learning (organ systems and common illnesses)	Biopsychosocial (horizontal strands) learning
Immunology	Communications
Oncology and hematology	Cultural competency
Microbiology and infectious disease	Ethics &and professionalism
Musculoskeletal medicine	Epidemiology
Preventive medicine	Health policy
Pulmonary disease	Life cycle – aging
Cardiovascular disease	Life cycle – pediatrics
Renal and genitourinary disease	Nutrition
Gastroenterological disease	Patient safety
Endocrine disease	Pain
Dermatologic disease	

**Fig. 1:**
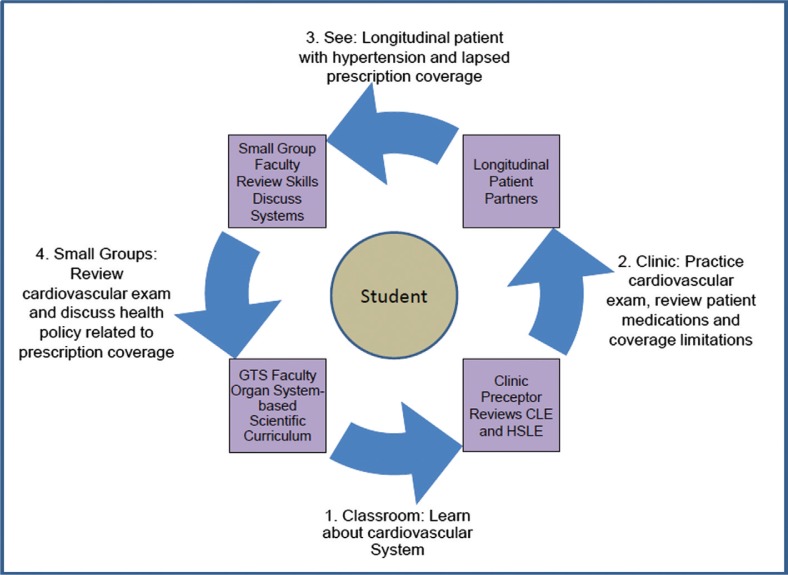
Integration in the longitudinal ambulatory clerkship. This figure demonstrated the integration of clinical and biopsychosocial learning in the LAC. If a student were learning about cardiovascular disease in classroom work, they might see a patient with hypertension and practice a cardiovascular exam on a patient they are following longitudinally for their clinical learning event (CLE). They might observe that poor control of blood pressure is related to failure to take medications due to a lapse in insurance coverage for their biopsychosocial learning event (BLE).

A unique aspect of the LAC is integration of clinical learning with learning in behavioral and social sciences (biopsychosocial learning). The clinical curriculum is built around 11 organ systems taught in GTS and also includes sessions that formally teach clinical reasoning and counseling. The biopsychosocial curriculum is conceptualized around 10 ‘horizontal strands’ (Table 2) that reflect national priority domains for behavioral and social sciences teaching (5). These are integrated within the 4-year GTS curriculum (3); like clinical learning, experiential grounding for these concepts in the first year is provided in the LAC. During each patient visit, students are asked to identify clinical and biopsychosocial learning events. The clinical learning event (CLE) focuses on evaluation and assessment of an illness or chronic condition. The biopsychosocial learning event (BLE) asks students to observe the impact of behavioral or social issues on patient care and outcomes. Fig. 1 illustrates how the LAC integrates clinical and biopsychosocial learning with the longitudinal care of patients.

### Coordination and monitoring

Because LAC is a widespread community-based program, we developed systems to assist with communication and monitoring. Weekly emails to our 120 student–faculty pairs remind preceptors about the scientific curriculum and review weekly learning objectives. To assure students stay on track with learning and assignments, they enter patient visits in a web-based patient log (e-log) and upload assignments into an electronic learning portfolio (e-portfolio). Clerkship administration tracks assignment completion, clinic assignments, and coordinates passport activities and transportation issues.

### Clinical learning

The bulk of CS learning occurs in the 24 clinic sessions as students see patients and practice interviewing, examination, documentation, and presentation skills with supervision from longitudinal preceptors. In addition to identifying a CLE and tracking this in their e-log, students complete five formal case write-ups that are uploaded into the e-portfolio and graded by faculty. A template was developed to assure that students adhered to a standard medical documentation format and understood content of standard elements.

### Biopsychosocial learning

The biopsychosocial learning occurs in concert with CS learning the 24 clinic sessions as students see patients and attend to the non-clinical factors that impact health and wellness. In addition to identifying a BLE and tracking this in their e-log, students are required to generate one written reflection for each of the 10 horizontal strands to help them consolidate thoughts and learning. Students may use a structured reflection template or may write an unstructured reflection. Reflections are uploaded to their e-portfolio.

### Grading and evaluation

The LC is graded using the four-level scheme used for all other clerkships in our institution (honors, high pass, pass, and fail). Students must complete all required clinic sessions, lectures and small group sessions to pass. Knowledge is evaluated by two written examinations, one at the end of each semester. CS are evaluated using graded examinations of two standardized patients at the end of the clerkship (6). In addition, preceptors evaluate student performance at the end of each of the clerkship's two semesters using a standard template with anchors. Grades are based on performance on written assignments, standardized patients, written examinations, and preceptor evaluations.

## Results: implementation, evaluation, and impact

Since its implementation in August 2009, 223 learners have completed the clerkship: 124 in its transitional one-semester class (LC1, August through December 2009) and 119 in its first complete two-semester class (LC2, January through December 2010). Evaluations are shown in Table 3. The transitional year was challenging: software and tracking systems were new; students were new to the curriculum and had not completed the intense CF course; community faculty were new to the clerkship, most faculty were new to clinical teaching; and clerkship administration was new. During LC1 we completed several cycles of feedback, and modified the clerkship and curriculum prior to implementing LC2. Between the two classes, there was significant improvement in the number of students who agreed or strongly agreed they achieved learning objectives (60.1 to 77.2%), and who rated the clerkship as very good or excellent (53.2 to 82.5%). According to student evaluations, the number of patients personally interviewed or examined during each clinic session increased from an average of 2.05 in LC1 to 2.67 patients per session in LC2, with total patient encounters including shadowing experience increasing from 4.85 to 5.60 patients per session. During LC2, the number of patients seen for repeat visits increased from 5.0% in the first semester to 9.3% in the second semester, which correlates to an average of 2.4 longitudinal patient visits per student; 96 of 119 students saw at least one patient more than once. Data from e-logs corroborated that students had learning experience in all clinical curriculum domains, with infectious illness cases most commonly reported. Communication and pain were the most frequently tracked biopsychosocial learning areas.

**Table 3:** Comparison of student evaluations of LC1 and LC2

	% agreed or strongly agreed	
	
	LC1 (124 respondents, 100% response)	LC2 (114 respondents, 97.4% response)
I received clear learning objectives for the clerkship	36.3	71.9
My performance was assessed against the learning objectives	32.2	62.9
I had the opportunity to follow a variety of different patients	65.3	71.9
My attending faculty members were adequately involved in teaching	77.2	91.2
I received helpful feedback on my performance during this clerkship	66.9	73.6
Faculty consistently modeled professionalism during this clerkship	87.1	92.9
Residents and fellows provided effective teaching during the clerkship	81.8	74.2
My overall learning objectives for this clerkship were met	60.2	77.2
Overall, how would you rate this clerkship?	53.2	82.5

Note: LC1 included 124 students in a transitional one-semester class (August through December 2009), and LC2 included 119 students in its first complete two-semester class (January through December 2010).

We are continuing to work on our teaching methodology. Our evaluations revealed that 97.3% of students agreed or strongly agreed that clinical practice sessions with ambulatory preceptors helped them meet the clerkships learning objectives, with similar high rates of agreement that PETA-exercises (88.4%) and formal case write-ups (85.1%) were useful. In contrast, only 17.5% agreed that written reflections were helpful and small groups were rated positively by only 12.3% of students.

## Discussion

The Longitudinal Ambulatory Clerkship was implemented as part of the new GTS curriculum, and reflects the broad goals of developing physicians who recognize each patient as a unique individual whose wellness is influenced by genetics and environment (3, 7). To incorporate pedagogical principals of medical education reform (1, 2), the LAC was developed to (1) provide early meaningful exposure to clinical medicine, (2) integrate clinical learning and experience with learning in the basic and social sciences, (3) provide learners with professional role models who share the broad functions of physicians, and (4) allow students to provide care to patients over a period of time. The combination of a formal clinical and biopsychosocial curriculum, infrastructure to demonstrate ongoing patient-related learning events, and focus on continuity practices in primary care settings allowed us to achieve our goals and provide a high quality, well-received experience for learners and faculty.

The LAC provides students with an opportunity to form long-term relationships with patients and faculty mentors, and offers an essential understanding of the benefits of strong physician–patient relationships and the longitudinal care of patients. We suspect that the improved evaluations between LC1 and LC2 are related to the longer duration of the clerkship and to development of our faculty as they gained experience with our curriculum and students. Learner satisfaction is closely related to quality of teachers (8, 9) so ongoing development and recognition of our new faculty will be key to the ongoing success of this clerkship. We are working to solidify the relationships of part-time faculty with the medical school by providing access to webinars and the CME courses and by including them in our teaching and advising communities. The success of these efforts is supported by positive faculty evaluations and high retention rates (89%) of faculty between LC1 and LC2.

Additional work is needed to increase satisfaction with reflective writing exercises and small group sessions. Reflection has been shown to promote lifelong learning and concept synthesis (10, 11). To help students organize their experience around horizontal strand concepts, we included written reflections in the biopsychosocial curriculum as learning exercises akin to the case write-ups in the clinical curriculum. However, we read only about one-fifth of reflections and did not have criteria for grading them, while we read and graded all case write-ups. Small groups served as forums for discussing reflections in the context of each horizontal strand. While students often shared write-ups with preceptors, they rarely shared reflections that sometimes questioned preceptor practice or decisions. While lower levels of satisfaction with these exercises could be related to a perception that biopsychosocial learning is less important than clinical leaning, we suspect that different approaches to review, grading, and discussion affected the perceived value of the exercises. We are working to orient students better to purpose of reflective writing, provide higher quality feedback to students, develop a formative dialoge with preceptors, and more objectively identify how well these exercises assist with concept mastery.

Other medical schools have implemented LCs in their third- and fourth-year curricula (12, 13), but we know of only a few that include these in the first-year curriculum. Whether or not this clerkship will improve our students’ performance on standardized exams, improve performance in clerkships or residency, or affect career choice compared to the old curriculum is yet to be determined. While start-up was challenging, the combination of faculty retention and positive student evaluations suggests this is a promising model of a clinically meaningful longitudinal experience in ambulatory medicine and primary care.

## References

[1] Irby DM, Cooke M, O'Brien BC (2010). Calls for reform of medical education by the Carnegie Foundation for the Advancement of Teaching: 1910 and 2010. Acad Med.

[2] Cooke M, Irby DM, Sullivan W, Ludmerer KM (2006). American medical education 100 years after the Flexner report. N Engl J Med.

[3] Wiener CM, Thomas PA, Goodspeed E, Valle D, Nichols DG (2010). Genes to Society’ – the logic and process of the new curriculum for the Johns Hopkins University School of Medicine. Acad Med.

[4] Stewart RW, Barker AR, Shochet RB, Wright SM (2007). The new and improved learning community at Johns Hopkins University School of Medicine resembles that at Hogwarts School of Witchcraft and Wizardry. Med Teach.

[5] Cuff PA, Vanselow NA, Institute of Medicine (2004). Committee on behavioral and social sciences in medical school curricula. Improving medical education: enhancing the behavioral and social science content of medical school curricula.

[6] Thomas PA, Shatzer JH (2000). Standardized patient assessment of ambulatory clerks: effect of timing and order of the clerkship. Teach Learn Med.

[7] Childs B, Wiener C, Valle D (2005). A science of the individual: implications for a medical school curriculum. Annu Rev Genomics Hum Genet.

[8] Sisson SD, Boonyasai R, Baker-Genaw K, Silverstein J (2007). Continuity clinic satisfaction and valuation in residency training. J Gen Intern Med.

[9] Serwint JR, Feigelman S, Dumont-Driscoll M, Collins R, Zhan M, Kittredge D (2004). CORNET Investigators. Factors associated with resident satisfaction with their continuity experience. Ambul Pediatr.

[10] Mann K, Gordon J, MacLeod A (2009). Reflection and reflective practice in health professions education: a systematic review. Adv Health Sci Educ Theory Pract.

[11] Sobral DT (2001). Medical students’ reflection in learning in relation to approaches to study and academic achievement. Med Teach.

[12] Ogur B, Hirsh D, Krupat E, Bor D (2007). The Harvard Medical School-Cambridge integrated clerkship: an innovative model of clinical education. Acad Med.

[13] Norris TE, Schaad DC, DeWitt D, Ogur B, Hunt DD, Consortium of Longitudinal Integrated Clerkships (2009). Longitudinal integrated clerkships for medical students: an innovation adopted by medical schools in Australia, Canada, South Africa, and the United States. Acad Med.

